# Nitrogen-doped porous carbon from biomass with superior catalytic performance for acetylene hydrochlorination†

**DOI:** 10.1039/d0ra00475h

**Published:** 2020-04-09

**Authors:** Zhaobing Shen, Yue Liu, Yejun Han, Yejun Qin, Jinhua Li, Ping Xing, Biao Jiang

**Affiliations:** Shanghai Green Chemical Engineering Research Centre, Shanghai Institute of Organic Chemistry No. 345 Lingling Road Shanghai P. R. China jiangb@sioc.ac.cn; Green Chemical Engineering Research Centre, Shanghai Advanced Research Institute, Chinese Academy of Sciences No. 99 Haike Road, Zhangjiang Hi-Tech Park, Pudong Shanghai P. R. China

## Abstract

Acetylene hydrochlorination is an important aspect of the industrial synthesis of polyvinyl chloride, but it requires a toxic mercury chloride catalyst. Here we report a green, highly efficient and low cost nitrogen-doped soybean meal carbon (SBMC) catalyst obtained from the simple carbonization of biomass soybean meal (SBM) in the presence of zinc chloride. This material exhibits excellent catalytic performance during acetylene hydrochlorination, with an initial acetylene conversion greater than 99% and 98% selectivity for vinyl chloride at 200 °C over 110 h. Analyses by X-ray photoelectron spectroscopy and temperature programmed desorption as well as catalytic activity evaluations show that pyridinic species are the active sites for hydrogen chloride, while pyrrolic N species are the main active sites for acetylene. An analysis of charge calculations based on model catalysts further indicates that the activity of pyrrolic N species essentially determines the performance of the SBMC catalyst. This investigation of the mechanism of acetylene hydrochlorination over SBMC confirms that such nitrogen-doped catalysts have two different active sites for the adsorption and activation of hydrogen chloride and acetylene molecules. This mechanism is different from that associated with metal chloride catalysts such as HgCl_2_. This SBMC catalyst is a potential alternative to HgCl_2_@AC catalysts for vinyl chloride synthesis and suggests a new means of designing carbon catalysts with basic surfaces for acetylene hydrochlorination.

## Abstract 2

A green, highly efficient and low-cost nitrogen-doped soybean metal carbon (SBMC) catalyst obtained from the simple carbonization of biomass soybean meal (SBM) in the presence of zinc chloride.

## Graphical abstract

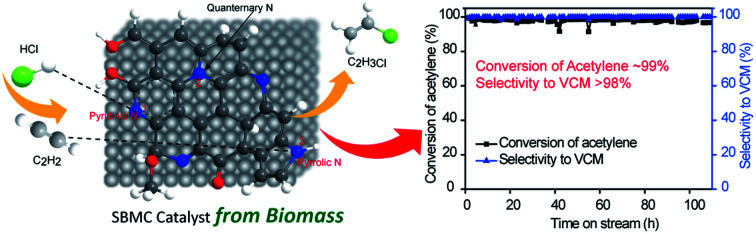

## Introduction

Polyvinyl chloride (PVC) is one of the most widely used plastics, with close to 41 million tons produced in 2016 and an annual growth in production of 5%.^1,2^ In developing countries with rich coal reserves, acetylene hydrochlorination catalysed by mercuric chloride (HgCl_2_@AC) is the primary process used to synthesize the vinyl chloride monomer (VCM).^2,3^ However, mercury is a potent toxin capable of causing cell death, brain damage and birth defects.^4–6^ Hence, more than 140 nations have agreed to a legally binding treaty related to the reduction of mercury release and use, signed in October 2013.^7,8^ The global Minamata Convention on Mercury^9^ came into effect on August 16, 2017 and has exerted significant pressure on the industrial production of acetylene-based PVC. Consequently, the research and development of mercury-free catalysts for PVC production and the sustainable development of the chlor-alkali industry are of considerable interest, and there has been extensive research in this field. Pioneering work and systematic studies performed by Hutchings and co-workers involving more than 30 metal chlorides showed that AuCl_3_ may be an alternative to HgCl_2_ based on its high activity.^10–12^ However, the cost of the noble metal gold and the rapid deactivation of this gold-based catalyst limit its practical applications.^13^

Carbon-based materials, particularly nitrogen-doped carbon, have recently received much attention as possible metal-free catalysts for acetylene hydrochlorination.^1,2,14–21^ Bao and co-workers reported that a nitrogen-doped SiC by vapor deposition method exhibited good catalytic performance for acetylene hydrochlorination, with an acetylene conversion 80% and selectivity to VCM over 98% at 200 °C. Furthermore, they stated that carbon atoms bonded with pyrrolic N species were the active sites by DFT and experiments.^2^ Dai, Zhu and co-workers did widely studies of mercury-free catalysts and reported several nitrogen-doped carbon catalysts supported on AC, using cyanamide, melamine or aniline as doping precursors. These nitrogen-doped carbon catalysts exhibited improved catalytic activity compared with AC undoped. Furthermore, they demonstrated that pyridinic N and pyrrolic N maybe the active sites for hydrogen chloride and acetylene, and pore character effect on catalysis by experiment and DFT.^17,19,20,22–25^ Li and co-workers also reported many works of mercury-free catalyst, including nitrogen-doped carbon catalyst for acetylene hydrochlorination.^26^ Jiang and co-workers reported the catalytic coupling reaction of acetylene and ethylene dichloride to synthesize VCM using nitrogen-doped activated carbon (AC) as the catalyst. Their results showed that nitrogen-doped AC not only catalyses acetylene hydrochlorination but also promote the dehydrochlorination of dichloroethane.^1^ In recent years, MOF and MOF-derived nitrogen-doped carbon materials were widely studied in supercapacitor and catalysis, especially for enhanced ORR performance.^27,28^ Moreover, Li *et al.* reported a number of metal–organic framework-derived nitrogen-doped carbon catalysts for acetylene hydrochlorination, and they demonstrated improved catalytic performance compared with AC.^16,29–31^

Nitrogen-doped carbon as a metal-free catalyst exhibits its unique character including green, low-cost and good catalytic performance, which has been a research hotspot in mercury-free catalyst for acetylene hydrochlorination. Even so, the study of nitrogen-doped carbon catalyst is still at an early stage, and is presently not sufficient for use in the industrial production of VCM. The prominent issue of nitrogen-doped carbon catalyst lies in its lower acetylene conversion and higher reaction temperature than metal catalyst, which motivates the further study for it. Furthermore, the manufacturing of such nitrogen-doped carbon materials usually involves the following characters, such as a complex synthesis processing, a nitrogen from external sources or the use of non-renewable carbon and nitrogen precursors. Thus, it is imperative to develop and scale up new methods of synthesizing highly efficient nitrogen-doped carbon catalysts through facile, green and low-cost routes. It would be even more desirable to develop these materials based on sustainable biomass resources.

Soybean meal (SBM) is a by-product of soybean oil extraction, and global SBM production is presently over one hundred million tons. Thus, this material represents a readily available, sustainable and inexpensive biomass. The crude protein content of soybean meal is as high as 30–50%, suggesting significant potential as an excellent precursor for the synthesis of nitrogen-doped carbon materials.
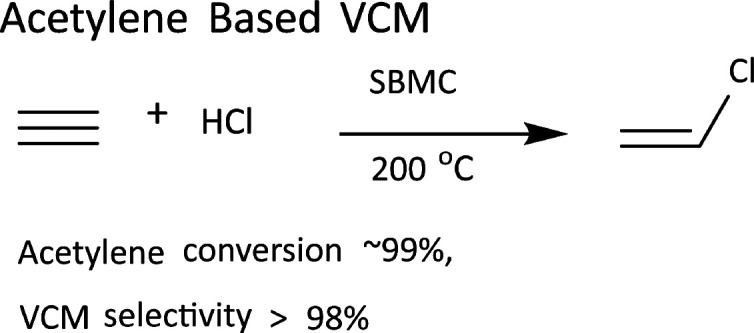


**Fig. 1:**
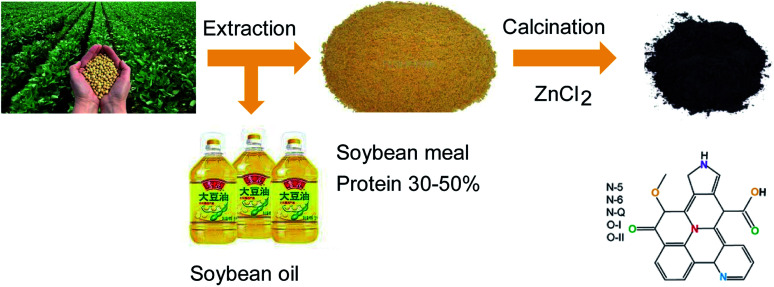
The synthesis of nitrogen-doped porous carbon from soybean meal bio-waste.^32^

On this basis, the present work used SBM as a precursor to prepare nitrogen-doped porous SBM carbon (abbreviation SBMC) by a facile pyrolysis process (Fig. 1). The resulting SBMC exhibits superior catalytic performance, with an initial acetylene conversion of approximately 99% and greater than 98% selectivity for VCM at 200 °C. Because SBM already contains a high level of nitrogen, preparation of the SBMC does not require an external nitrogen source, but rather is based simply on calcination of the SBM with ZnCl_2_ as dehydrating agent and pore former. These SBMC catalysts show good stability, with acetylene conversions from 99% to 97% during a 110 h test at 200 °C. This material therefore has excellent potential as an alternative to HgCl_2_ as a catalyst for the acetylene-based VCM synthesis process. Moreover, on the basis of the previous works,^2,19,20^ this present work demonstrated that nitrogen-doped mainly contributed to the catalytic activity of SBMC and nitrogen-doped carbon by the experiments, but not oxygen-doped or defective carbon without nitrogen. Subsequently, the combination of catalytic reaction, XPS, TPD and charge calculation detailly stated that pyridinic N tended to adsorb and activate hydrogen chloride attributing to the alkalic and electron donor of pyridinic N, and pyrrolic N preferred to adsorb and activate acetylene due to the positive charge on the pyrrolic N. This study inherits the previous works, further understanding the catalytic nature of nitrogen-doped carbon for acetylene hydrochlorination by experiments in detail.

## Results and discussion

### Characterization of SBMC

The SBMC (Fig. 1) was prepared by mixture of ZnCl_2_ and SBM, which calcined at 500, 600, 700 or 800 °C. ZnCl_2_ was removed by washing water. The elemental compositions of the original SBM and the SBMC specimens were determined by combustion-based elemental analysis, X-ray photoelectron spectroscopy (XPS) and inductively coupled plasma mass spectrometry (ICP-MS). The results were summarized in Table 1. It can be seen that the SBMC yields at the different carbonization temperatures were all high (over 30%).^33^ It is also apparent that there were no variations in the elemental compositions of the SBMC processed at the different temperatures. These results indicate that the carbonization of the SBM was essentially complete even when using the relatively low temperature of 500 °C for 2 h, as a result of the presence of ZnCl_2_. Comparing the elemental analysis data for the SBM and the SBMC-600, it is apparent that more H and O was lost during calcination as compared with C and N. This difference is attributed to the dehydration reaction that occurs when using ZnCl_2_ as an activating agent at 600 °C. The loss of C, H, N and O facilitated the formation of a porous/defective structure in the SBM during carbonization. The XPS, used to measure the atoms on the surface of material, results showed higher N/C ratio and lower O/C ratio on the surface of SBMC than the data by the combustion elemental analysis measurement, which indicated that the dehydration reaction was higher efficient on the surface of SBM than in the interior when using ZnCl_2_ in the carbonization process. The higher content of N/C showed that there was much more nitrogen-doped carbon and defective structure on the surface of SBMC, more contributing to the catalytic performance. In addition, the N/C and O/C ratios in the SBMC specimens by XPS were almost constant, and thus they were evidently unaffected by variations in the carbonization temperature 500–800 °C. The ICP-MS results demonstrate the presence of trace amounts of metal ion in the SBMC samples, even after repeated washing of the crude products with dilute hydrochloric acid and deionized water with the intent of reducing the negative effects of ions such as Ca^2+^ and K^+^. The residual Zn^2+^ from the activation agent was present only at very low levels.

**Table 1:** Yields of the SBMC samples and the elemental compositions of the SBM and SBMC

Sample	Yield^a^ (%)	Chemical composition^b^^,^^c^ (at%)				Mole ratio^d^		Chemical composition^e^ (ppm)						
		C	H	N	O	N/C	O/C	Ca	K	Mg	Zn	Al	Fe	Na
SBM	—	42.4	6.3	7.2	37.2	0.17	0.88	129.6	411.6	93.1	2.4	8.7	12.4	2.6
SBMC-500	30.8	63.4	3.8	6.8	15.6	0.11	0.25	1.6	0.3	0.2	62.8	0.4	0.6	0.2
SBMC-600	34.8	72.6	3.2	6.9	7.8	0.10	0.11	0.9	0.3	0.2	73.7	1.1	1.1	0.2
SBMC-700	31.8	63.5	3.3	7.0	16.4	0.11	0.26	2.2	0.4	0.3	83.8	1.3	2.6	0.4
SBMC-800	33.1	65.0	3.3	6.5	14.7	0.10	0.23	1.9	2.0	0.8	127.6	2.6	9.5	0.3
SBMC-500	—	72.0	—	12.1	15.9	0.14	0.17	—	—	—	—	—	—	—
SBMC-600	—	77.3	—	8.9	13.7	0.10	0.13	—	—	—	—	—	—	—
SBMC-700	—	74.2	—	12.6	13.2	0.15	0.13	—	—	—	—	—	—	—
SBMC-800	—	75.7	—	11.3	13.0	0.13	0.13	—	—	—	—	—	—	—
Loss of SBMC-600^f^	—	40.4	82.3	26.5	92.7			—	—	—	—	—	—	—

a Calculated from the mass ratio of the obtained carbon to its precursor.

b Measured by combustion elemental analyses, line 1–line 5.

c Calculated using the XPS data for C, N and O, line 6–line 9.

d Calculated by ratioing moles of N and O to C.

e Based on data from ICP-MS.

f Calculated from the chemical composition data for SBM and SBMC-600.

**Fig. 2:**
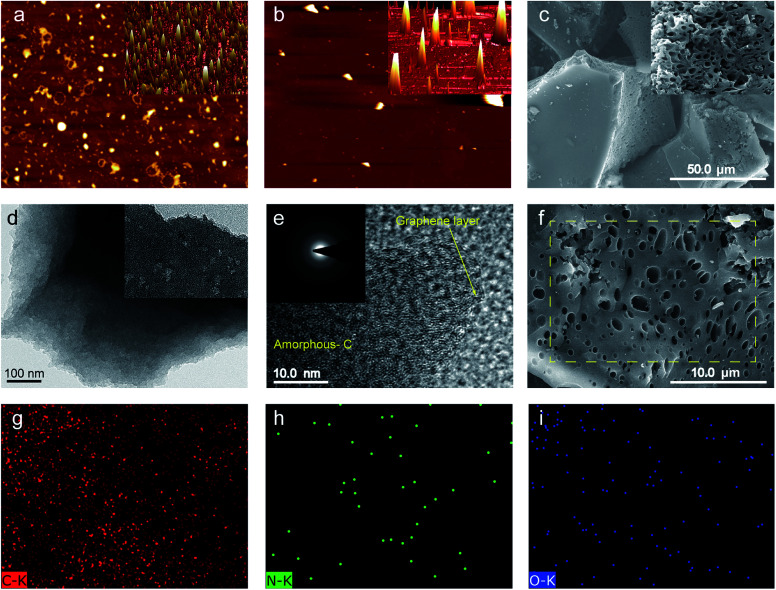
The 2D AFM images of (a) the SBM (inset: 3D image) and (b) the SBMC-600 (inset: 3D image). (c) SEM image of the SBMC-600 (inset: SEM image). (d) TEM image of the SBMC-600 (inset: TEM image). (e) High-resolution TEM image of the SBMC-600 (inset: SAED pattern). (f) High-powered SEM image of the SBMC-600 and the corresponding EDX maps for (g) C, (h) N and (i) O.

The general structures, sizes and morphologies of the SBMC specimens were assessed using atomic force microscopy (AFM), scanning electron microscopy (SEM) and high-resolution transmission electron microscopy (HRTEM), as shown in Fig. 2. The AFM images demonstrate that the SBM and SBMC-600 were comprised of irregularly shaped particles, indicating that carbonization had no effect on the morphology. The 3D images in this figure also show irregular shapes for both materials (insets to Fig. 2a and b). The SEM image of the SBMC indicates irregularly shaped particles with smooth surfaces (Fig. 2c) and a large number of continuous mesopores and macropores along the cross section (inset to Fig. 2c and f). The pores had irregular shapes and diameters ranging from ∼100 nm to ∼2 μm. These pores can be attributed to various synergistic effects, including etching and swelling as well as the template effect of the ZnCl_2_. Zinc chloride could catalyze the dehydration and condensation reactions in SBM, and also acted as a pore former and template during the carbonation process, all contributing to the formation of porous carbon material. It was reported that the impregnation of ZnCl_2_ played an important role in increasing specific surface area and generation of micropores. The impregnation of ZnCl_2_ acts as a skeleton and template and occupies a volume, inhibiting the contraction of the particle during the carbonization, which can leave porosity structure after being washed off with deionized water.^34,35^ In addition, the energy dispersive X-ray spectroscopy (EDX) maps (Fig. 2g–i) indicate uniform distributions of carbon, nitrogen and oxygen throughout the SBMC-600. The SBMC microstructure was further analysed by TEM (Fig. 2d) and HRTEM (Fig. 2e). The resulting images show that the material contained both amorphous carbon and graphite, with numerous nanopores. The corresponding selected area electron diffraction (SAED) pattern (inset to Fig. 2e) exhibits diffraction rings that confirm the amorphous nature of this material, which had a turbostratic structure.

**Fig. 3:**
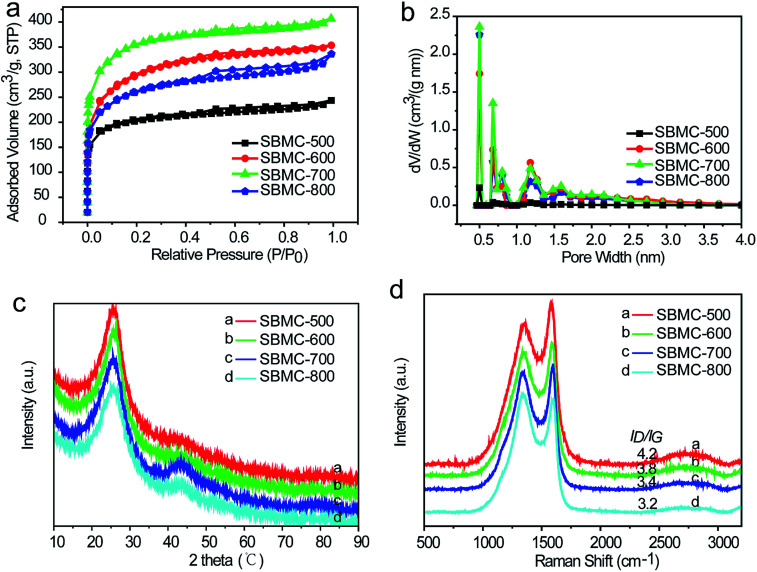
The structural characterization of SBMC specimens synthesized by carbonization at different temperatures. (a) N_2_ adsorption–desorption isotherms, (b) pore size distributions, (c) X-ray diffraction patterns and (d) Raman spectra.

N_2_ adsorption/desorption isotherms were obtained to characterize the pore structures (Fig. 3a and b). Fig. 3a demonstrates that the SBMC materials generated a combination of types I and IV isotherms according to the IUPAC classification system. High adsorption capacities at low relative pressures (*P*/*P*_0_ < 0.1) were observed, indicating the presence of a significant quantity of micropores. Type H4 hysteresis loops caused by capillary condensation at *P*/*P*_0_ values ranging from 0.4 to 1 were also observed, suggesting the presence of mesopores as well. Notably, the adsorption capacity increased with increases in the carbonization temperature from 500 to 700 °C but then decreased at 800 °C. These results show that the SBMC synthesized at a relatively high temperature had a distinct surface area and volume. The properties at intermediate temperatures likely resulted from the accelerated reaction between the carbon precursor and the ZnCl_2_, leading to higher porosity and enlarged pores. In contrast, the carbon skeleton was destroyed and collapsed by the action of the ZnCl_2_ at 800 °C.^36,37^ The pore size distributions of these materials are summarized in Fig. 3b. It is evident that micropores and a smaller quantity of mesopores (with sizes ranging from 0.5 to 3.5 nm) were present, consistent with the results in Fig. 2a. Table 2 summarizes the textural parameters of the SBMC. As the carbonization temperature was increased from 500 through 600 to 700 °C, the specific surface area and pore volume increased dramatically, from 739 to 1038 and 1124 m^2^ g^−1^ and from 0.31 to 0.46 to 0.52 cm^3^ g^−1^, respectively. However, the specific surface area and pore volume all decreased when the temperature was raised to 800 °C. These variations in textural parameters with carbonization temperature are in agreement with the adsorption capacity data (Fig. 3a and b). The observed increases in specific surface area can be primarily ascribed to the formation of mesopores by the ZnCl_2_. The fraction of the specific surface area contributed by mesopores increased from 0.28 to 0.51, 0.28 and 0.45 as the temperature was increased. Similarly, the contributions of the mesopore volume to the total pore volume increased from 0.29 to 0.52, 0.35 and 0.48 with increasing temperature.

**Table 2:** Textural parameters of the SBMC

Sample	*S*_BET_ (m^2^ g^−1^)				Pore volume (cm^3^ g^−1^)				
	Total	Micro	Meso	Ratio^a^	Total	Micro	Meso	Ratio^b^	*D*_p_ c (nm)
SBMC-500	739	530	209	0.28	0.31	0.22	0.09	0.29	3.1
SBMC-600	1038	505	533	0.51	0.46	0.22	0.24	0.52	2.6
SBMC-700	1124	811	473	0.28	0.52	0.34	0.18	0.35	2.7
SBMC-800	938	518	420	0.45	0.42	0.22	0.20	0.48	3.2
SBM-800^d^	19.9	15.1	4.8		0.013	0.006	0.007		12.3

a Ratio of mesopore area to total area.

b Ratio of mesopore volume to total volume.

c Average pore diameter.

d Without ZnCl_2_.

X-ray diffraction (XRD) patterns were used to investigate the structures of the SBMC prepared at different carbonization temperatures (Fig. 3c). Two diffraction peaks were observed at ∼25.5° and ∼43.5° corresponding to the (002) and (100) planes of graphite, respectively. It was notable that the diffraction peak at ∼25.5° was relatively sharp and narrow, indicating a certain degree of graphitization of the SBMC synthesized at 500–800 °C. As the temperature was increased from 600 through 700 to 800 °C, peaks appeared at ∼43.5°, demonstrating that (100) graphite planes were formed in the SBMC. However, the resulting SBMC produced at all carbonization temperatures generally had an amorphous structure with a relatively low degree of graphitization, in agreement with the HRTEM images in Fig. 2e. This result can likely be attributed to the local lattice distortion caused by heteroatom (N and O) doping. It was also observed that the abundant pores formed by the activation reaction not only led to a breakdown of aligned structural domains in the SBMC, but also disturbed the stacking periodicity of the graphitic carbon structure.^38,39^

The Raman spectra of the SBMC are shown in Fig. 3d. The peaks at 1353 cm^−1^ (the D band) and 1585 cm^−1^ (the G band) reflect disordered sp^3^ C atoms/defective graphitic structures and ordered carbon structures with sp^2^ C atoms/crystalline graphite, respectively, and can be used to confirm the amorphous structure of the SBMC.^40^ The degree of graphitization is usually estimated based on the ratio of the D-band to the G-band (*I*_D_/*I*_G_), although this ratio is also significantly affected by both the nitrogen content and the presence of coupled molecular pores and edge terminations.^41^ With increases in the carbonization temperature, the value of *I*_D_/*I*_G_ gradually became lower (Fig. 3d), implying increased graphitization of the SBMC, consistent with the XRD patterns (Fig. 3c).

**Fig. 4:**
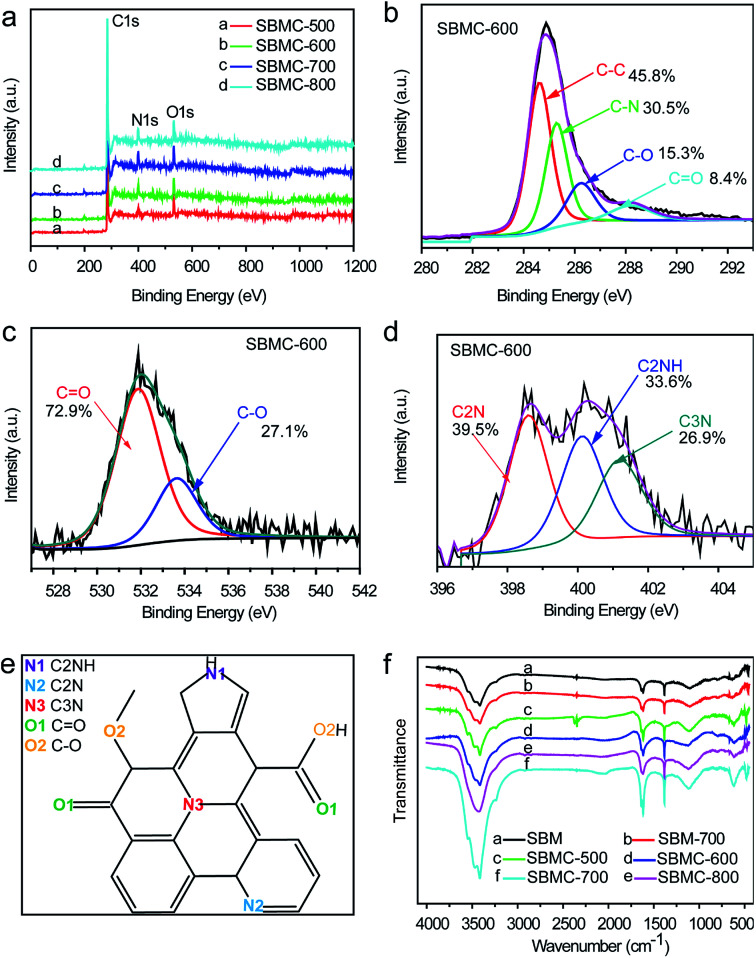
(a) XPS survey spectra of the SBMC, the high-resolution (b) C 1s, (c) O 1s and (d) N 1s spectra of the SBMC-600, (e) the molecular structure of the SBMC and (f) the FTIR spectra of the SBMC.

XPS data were acquired to assess the surface elemental compositions and configurations of doped heteroatoms in the SBMC (Fig. 4). Fig. 4a demonstrates three characteristic peaks observed at ∼284, ∼400 and ∼531 eV, which can be assigned to C 1s, N 1s and O 1s signals, respectively. These results indicate that nitrogen and oxygen atoms were successfully doped into the carbon-based structure of the SBMC, to a greater extent than observed in the case of nitrogen-doped carbon catalysts (NAC) by post-treatment organic nitrogen processing.^1,2,17,19^ The high-resolution XPS C 1s spectra of the SBMC-600 (Fig. 4b) can be deconvoluted into four individual peaks corresponding to C–C (284.5 eV), C–N (285.2 eV), C–O (286.3 eV) and C=O (288.3 eV), further confirming the successful incorporation of nitrogen and oxygen into the carbon framework, consistent with the results shown in Fig. 4a.^1^ The high-resolution O 1s spectrum in Fig. 4c clearly establishes the presence of several oxygen-based groups, including quinone-type C=O (O-I) and phenol-type C–OH (O-II), which might have a positive effect on catalytic performance during acetylene hydrochlorination.^13,42^ The high-resolution N 1s spectrum of the SBMC-600 was acquired to gain further insights into the extent of nitrogen doping. As shown in Fig. 4d, the deconvolution of the high-resolution N 1s spectrum produced three peaks corresponding to pyridinic N (398.6 eV), pyrrolic N (400.2 eV) and graphitic N (401.2 eV). The molecular structure of the SBMC suggested by these data is provided in Fig. 4e. Bao and co-workers previously reported that carbon atoms bond with pyrrolic N species to produce active sites for acetylene hydrochlorination, based on both experimental and theoretical studies.^2^ Dai, Jiang and other researchers have determined that pyridinic N and carbon atoms bonded to such species represent active sites, based on both experiments and density functional theory calculations.^1,19,20^

The Fourier transform infrared (FTIR) spectra of the SBM and the SBMC prepared at different carbonization temperatures are provided in Fig. 4f. After carbonization at 500–800 °C, the obtained SBMC exhibited very similar spectra to that of the raw SBM. This result suggests that the surface functional groups of the SBMC remained the same before and after carbonization. Both the SBMC and SBM exhibited characteristic O–H and N–H stretching vibration peaks at ∼3405 and ∼3285 cm^−1^ as well as a C–H stretching peak at ∼2900 cm^−1^. The characteristic absorption bands of aromatic CN heterocycles were also observed in the range of ∼1280 to ∼1605 cm^−1^.^43^ These data imply that the local structure of these carbon materials comprised CN units, consistent with the XPS analysis. Together, the data demonstrate the unique features of SBMC, such as high specific surface areas and pore volumes and significant heteroatom doping.

### Catalytic performance of SBMC

**Fig. 5:**
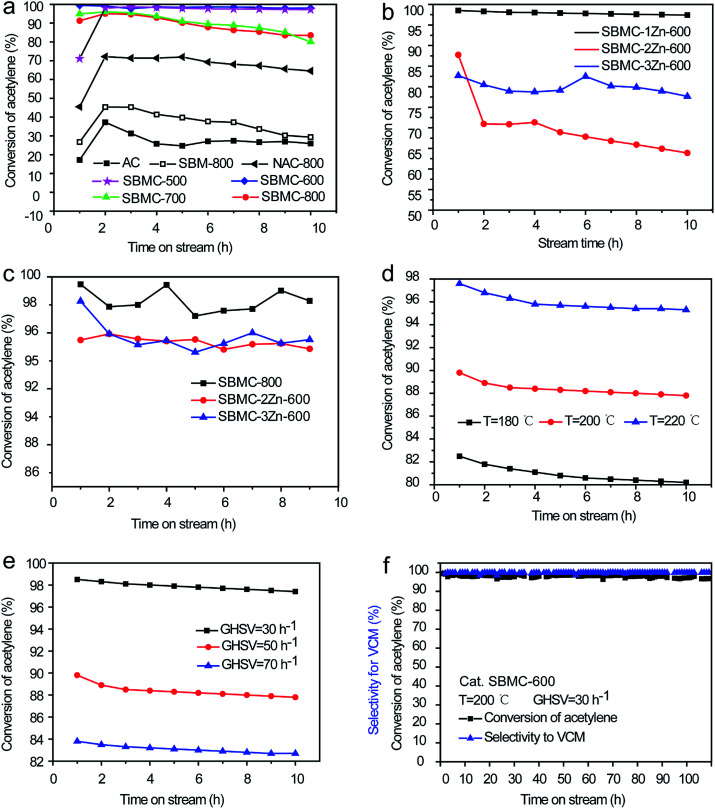
Catalytic performances of the SBMC as functions of (a) carbonization temperature, (b) dosage of ZnCl_2_, (c) residual ZnCl_2_, (d) reaction temperature, (e) gas hourly space velocity (GHSV) of C_2_H_2_ and (f) the data from long-term stability testing of the SBM-600. Reactions were carried out at 200 °C, atmospheric pressure, a space velocity of 1.0 mL g^−1^ min^−1^ (GHSV = 30 h^−1^) and HCl/C_2_H_2_ = 1.15/1.0 (volume ratio) unless otherwise stated. The selectivity for VCM was above 98% at all times.

The relationships between the catalytic activities of the SBMC and processing parameters including carbonization temperature, ZnCl_2_ concentration, reaction temperature and gas hourly space velocity (GHSV) of C_2_H_2_ were examined, as shown in Fig. 5a–e. The use of AC gave an acetylene conversion of just 30% at 200 °C, atmospheric pressure and a space velocity of 30 h^−1^, while SBM-800 was prepared by simply calcination of SBM at 800 °C without addition ZnCl_2_ and showed ∼40% conversion of acetylene under the same reaction conditions (Fig. 5a). Although the AC had a high specific surface area of ∼997 m^2^ g^−1^, it showed a lower acetylene conversion than the SBM-800, which had an area of only 19.9 m^2^ g^−1^ (Table 2). The superior performance of the latter material is attributed to the bonds between carbon and nitrogen species.^1,2,19,20^ As shown in Fig. 5a, the SBMC synthesized at 500 or 600 °C in conjunction with ZnCl_2_ exhibited excellent performance, giving an initial acetylene conversion of ∼99.5% at 200 °C, atmospheric pressure and a gas hourly space velocity of 30 h^−1^, without any obvious inactivation after 10 h. The SBMC obtained at 700 and 800 °C gave an initial acetylene conversion as high as ∼98% but showed reduced stability. As shown in Table 2, it was found that the data of the specific area and nitrogen contents of SBMC-700 and SBMC-800 was almost same as them of SBMC-500 and SBMC-600, which implied that the reduced stability of SBMC-700 and SBMC-800 had nothing to do with the catalysts themselves and carbonization temperature. Besides, Zhu *et al.* reported that nitrogen-doped carbon catalyst had excellent catalytic performance at 900–1100 °C, which further demonstrated that high carbonization temperature didn't have negative effect on the catalytic activity of nitrogen doped carbon.^23^ The above result implied that the other key factor of ZnCl_2_ residual might be negative for the catalytic performance. As shown in Table 1, the data of ZnCl_2_ residual in SBMC-700 and SBMC-800 was higher than that of SBMC-500 and SBMC-600. To demonstrate it, SBMC-800 was immersed in 10% (v/v) hydrochloric acid at 50 °C for 12 h, washed with deionized water then dried at 70 °C. As shown in Fig. 5c, the repeatedly washed SBMC-800 exhibited very excellent catalytic performance and stability, with a conversion of acetylene over 98% in 10 h, which strongly stated that the ZnCl_2_ residual was a critical parameter in preparation of nitrogen doped carbon catalysts. In fact, a high carbonization temperature has no negative influence on catalytic performance of SBMC, but it might make ZnCl_2_ difficult to remove completely. To compare to the SBMC, a material termed NAC-800 was prepared using AC and acrylamide as the carbon and nitrogen precursors, respectively, at a mass ratio of acrylamide to AC 1 : 1, using a post-treatment technology. The NAC-800 exhibited only 70% conversion of acetylene under the same reaction conditions, which was attributed to the high loss percentage and low dispersion of nitrogen atoms during the post-treatment process. Comparing the catalytic performances of the SBMC and the NAC-800 highlights the advantages of the combination of high carbon and nitrogen levels in the SBMC, which ensures improved nitrogen distribution and lower losses.

The effects of the ZnCl_2_ amount on the catalytic activities of the SBMC were investigated (Fig. 5b). It was found that the catalytic activity was significantly increased when using ZnCl_2_ as an activating agent and dehydrate agent (Fig. 5a). In a further set of experiments, materials termed SBMC-1Zn-600, SBMC-2Zn-600 and SBMC-3Zn-600 were prepared, with ZnCl_2_ to SBM mass ratios of 1.0, 2.0 and 3.0, respectively. As shown in Fig. 5b, increasing the level of ZnCl_2_ relative to the SBM decreased the catalytic activity. The SBMC-1Zn-600 gave an acetylene conversion greater than 97% and showed significant stability at 200 °C, while the SBMC-2Zn-600 and SBMC-3Zn-600 both showed poor catalytic activity and stability. There was no obvious correlation between ZnCl_2_ adding amount and catalytic performance, implying that traces of residual ZnCl_2_ might poison the SBMC. Similarly, the SBMC-800 exhibited a poisoning phenomenon (Fig. 5a) and was confirmed by the extra experiment (Fig. 5c). To confirm the effect of residual ZnCl_2_ on catalytic activity, samples of SBMC-2Zn-600, SBMC-3Zn-600 were washed by the same method as that of SBMC-800. As shown in Fig. 5c, the catalytic performance and stability of each material was obviously increased, indicating the toxicity of the residual ZnCl_2_. So, the suitable dosage of ZnCl_2_ is beneficial for the catalytic performance and preparation cost.

In contrast to mercuric chloride which is prone to sublimation under high temperature, *i.e.*, 200 or 220 °C, this catalyst is rather robust. It can be operated at an even higher temperature and space velocity, as shown in Fig. 5d and e, respectively. For example, at 220 °C and 50 h^−1^ the conversion of acetylene reached 95% and the selectivity to VCM remained above 98%. Furthermore, the SBMC-600 exhibited good stability, such that the conversion of acetylene only decreased slightly during a 110 h test at 200 °C, as demonstrated in Fig. 5f.

**Fig. 6:**
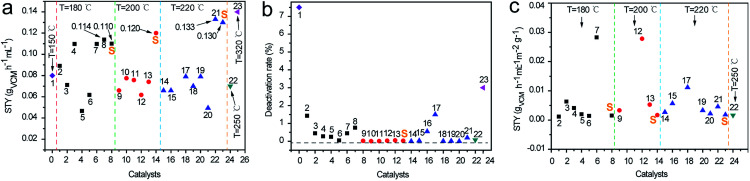
Comparison of this work and the previous works of the metal-free catalysts. (a) The space-time yield (STY) of VCM; (b) the deactivation rate of catalysts; (c) the STY normalized by the surface area and nitrogen contents. The numbers represent the previous works of the metal-free catalysts in the literatures and S denotes this work. 1,^18^ 2,^20^ 3,^17^ 4,^16^ 5,^44^ 6,^13^ 7,^45^ 8,^46^ 9,^2^ 10,^15^ 11,^47^ 12,^48^ 13,^49^ 14,^50^ 15,^51^ 16,^22^ 17,^23^ 18,^52^ 19,^53^ 20,^54^ 21,^24^ 22,^19^ and 23.^55^

To compare this work with the previous works in the literatures at the similar conditions, the space-time yield of VCM (STY_VCM_) were calculated in Fig. 6a. To get the reliable result, this work was carried out on the similar conditions as the literatures, *i.e.*, at 180, 200, and 220 °C. As shown in Fig. 6a, the value of STY_VCM_ in this work was higher than the most results from the references, which sated the superior catalytic performance of SBMC. But the value of STY_VCM_ in this work was little lower than TPPB@SAC and NC-2 in the literature. TPPB@SAC is a ionic liquid derived metal-free catalyst and NC-2 is a macroporous nitrogen-doped carbon catalyst. Furthermore, the deactivation rate of SBMC was compared with the works in the literatures in Fig. 6b. As shown in Fig. 6b, SBMC exhibited a very low deactivation rate, obviously lower than TPPB@SAC and NC-2. In a word, by contrast with STY_VCM_ and deactivation rate between SBMC and the works in the literatures, it was seen that SBMC exhibited superior catalytic performance for acetylene hydrochlorination.

To further understand the nature of the catalysts, the value of STY_VCM_ was normalized by the specific area and nitrogen contents (Fig. 6c). It was found that the value of normalized STY_VCM_ was almost lower than all the values from the literatures, which implied that the excellent catalytic performance of SBMC was mainly limited by the combined action of specific area and nitrogen content. This result also indicated that there was no extra catalytic active site on SBMC different from the common nitrogen doped carbon. In contrast, the STY_VCM_ value of TPPB@SAC and NC-2 further encourage us to optimize SBMC by introducing the innovative active sites and macroporous pores.

### Catalytic sites and acetylene hydrochlorination mechanism

In the past, there have been many efforts by various research groups to investigate the active sites of nitrogen-doped carbon materials during acetylene hydrochlorination, on the basis of both experimental and theoretical work. Bao *et al.* indicated that acetylene is only minimally absorbed on quaternary and pyridinic N sites because of the endothermic nature of the adsorption process. They suggested that carbon doped with pyrrolic N species could catalyse the hydrochlorination of acetylene.^2^ Dai *et al.* reported that hydrogen chloride is absorbed on g-C_3_N_4_*via* H atoms near pyridinic N atoms, while acetylene is adsorbed by carbon atoms bonded to pyridinic and graphitic N atoms.^20^ Wang *et al.* found that the active sites in PSAC-N comprise quaternary nitrogen atoms bonded between two 6-membered rings.^19^ Zhang *et al.* demonstrated that the order of importance of nitrogen species in acetylene hydrochlorination was: pyrrolic N > graphitic N > pyridinic N.^17^ However, the role of different N species in providing active sites is still being debated. It is critical to identify the active sites in SBMC during acetylene hydrochlorination to allow for further optimization of the catalyst. However, the coexistence of different N and O species in these materials makes it very difficult to assess their respective catalytic roles.

**Fig. 7:**
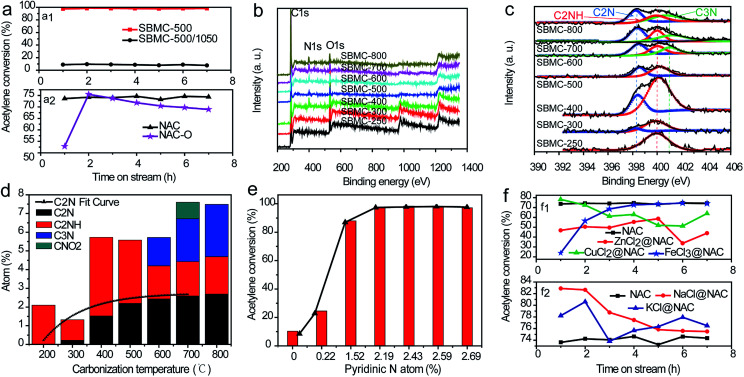
(a1) Effects of nitrogen atoms on catalytic activity and (a2) effects of oxygen atoms on catalytic activity. (b) XPS spectra of SBMC obtained at different carbonization temperatures. (c) Nitrogen atom types obtained at various carbonization temperatures. (d) The proportion of N atoms in different chemical states at different carbonization temperatures. (e) Acetylene conversion over SBMC carbonized at various temperatures. (f) Effects of metal chlorides on catalytic activity.

The effects of defective carbon on the catalytic performance of SBMC could also be important. The protein, crude fibre and nitrogen in SBM could all be degraded to produce low molecular substances through pyrolysis. These new compounds may subsequently reorganize to form nitrogen and oxygen co-doped carbon-based materials comprising the SBMC at elevated temperatures. In addition to the nitrogen and oxygen co-doped carbon skeleton, these materials might contain defective carbon phases resulting from the removal of nitrogen and oxygen atoms. Yao *et al.* reported a simple method to remove nitrogen atoms from nitrogen-doped carbon to obtain a defective phase that showed excellent performance during the oxygen reduction reaction (ORR) and hydrogen evolution reaction (HER).^56^ Li and Zhang *et al.* considered that the reaction mechanism during acetylene hydrochlorination was the same as that associated with the ORR.^17,20^ Based on these prior reports, the SBMC-500 was calcined at a high temperature of 1050 °C under a nitrogen atmosphere for 2 h, to produce a specimen termed SBMC-500/1050. As shown in Fig. 7a1, this material provided an acetylene conversion of ∼10% compared with 98% for the original catalyst. This contrast strongly indicates that the active sites in these SBMC for acetylene hydrochlorination were the nitrogen-doped carbon constructs, not the defective carbons without nitrogen atoms.

The effects of oxygen atoms on the catalytic activities of the SBMC were also assessed. Dai *et al.* elucidated the effect of oxygen-containing groups on the catalytic performances of nitrogen-doped graphene (N-G) and boron nitrogen co-doped graphene (B, N-G).^18^ Their work showed that the presence of oxygen atoms in the N-G and B, N-G catalysts decreased the adsorption of hydrogen chloride during the hydrochlorination of acetylene. According to the work,^13^ our own group prepared NAC and NAC-O catalysts using nitric acid. As shown in Fig. 7a2, the catalytic performance of the NAC-O during the hydrochlorination of acetylene was lower than that of the NAC catalyst because of the limited HCl adsorption onto active sites, consistent with the conclusion in ref. 18. Thus, the active sites on the SBMC are found in the nitrogen-doped carbon skeleton, not the oxygen-doped carbon skeleton or the defective carbon skeleton caused by the removal of nitrogen and oxygen atoms.

### The active sites for hydrogen chloride in the SBMC

It is well known that HgCl_2_ and AuCl_4_ on AC provide active sites for acetylene and hydrogen chloride, respectively, during acetylene hydrochlorination. Thus, the active sites on the present nitrogen-doped carbon catalyst, which differ from one another, were also assessed. Dai *et al.* reported that pyridinic nitrogen atoms serve as catalytically active sites for hydrogen chloride, while carbon atoms are adsorption sites for acetylene.^18,20^ Prior researchers did not perform separate studies concerning active sites for hydrogen chloride and acetylene, but rather focused on the overall effect of various types of nitrogen during the hydrochlorination of acetylene.^17,50^ We believe that the present analysis will therefore assist in designing improved nitrogen-doped catalysts for this reaction. According to prior reports,^1^ the chemical states of nitrogen atoms in nitrogen-doped catalysts are closely correlated with the carbonization temperature. XPS spectra were acquired to probe the proportions and chemical states of C, N, O and H atoms in the SBMC materials. As shown in Fig. 7b, three characteristic peaks were observed at ∼284, ∼400 and ∼531 eV, assigned to C 1s, N 1s and O 1s signals, respectively. With increases in the carbonization temperature, the proportion of nitrogen atom on the surface of the specimens gradually increased, from ∼2% at 250 °C to ∼7.5% at 700–800 °C, confirming that high temperatures favour the migration of nitrogen atoms from the bulk of the SBMC and the removal of H and O atoms. Furthermore, elevated temperatures obviously resulted in changes in the chemical states of nitrogen atoms (see Fig. 7c). Following treatment at 250 °C, only one characteristic peak, assigned to pyrrole N (C_2_NH) was observed in the spectrum. When the temperature was increased to 300 °C, some of this N was converted to pyridinic N (C_2_N), giving a proportion of ∼0.22%. Following processing at 400 °C, the pyrrole N (C_2_NH) was converted to pyridinic N (C_2_N) to a greater extent (∼1.52%). With treatment in the range of 500–800 °C, the proportion of pyridinic N gradually increased, along with an increase in graphitic N (C_3_N) (Fig. 7d). As shown in Fig. 7e, pyridinic N proportions in the range of 0–1.52% increased the extent of acetylene conversion to greater than 88.6%. This result suggests that pyridinic N atoms serve as critical active sites for acetylene hydrochlorination. As reported in ref. 1, pyridinic N contributes one p electron to the aromatic π electron system and also has a lone electron pair in the plane of the carbon matrix that increases the electron donor properties of the catalyst. The introduction of pyridinic-type N species into the carbon network adds basicity, such that the material can be used as a solid base catalyst. Research has shown that hydrogen chloride is easily adsorbed onto pyridinic N based on theoretical calculations.^20^ To further demonstrate the function of pyridinic N atoms as active sites for hydrogen chloride, we prepared NAC loaded with ZnCl_2_, CuCl_2_ and FeCl_3_, termed ZnCl_2_@NAC, CuCl_2_@NAC and FeCl_3_@NAC, respectively. As shown in Fig. 7f1, the acetylene conversions of the ZnCl_2_@NAC, CuCl_2_@NAC and FeCl_3_@NAC catalysts were all lower than that of NAC, because the Lewis acids occupied the pyridinic N sites of the NAC catalysts, inhibiting hydrogen chloride adsorption. These experimental results strongly suggest that the pyridinic N atoms were the active sites for the adsorption and activation of hydrogen chloride, which is the rate-determining step for acetylene hydrochlorination. We also prepared two catalysts with alkali salts (NaCl and KCl) loaded on NAC, denoted as NaCl@NAC and KCl@NAC. As shown in Fig. 7f2, the acetylene conversions of the NaCl@NAC and KCl@NAC were both higher than that of the bare NAC catalyst. This result shows that the alkali chlorides not only did not occupy the pyridinic N sites, but also provided active sites for adsorption of hydrogen chloride. Thus, the pyridinic N atoms in the nitrogen-doped carbon catalyst were the critical active sites for the adsorption and activation of hydrogen chloride during acetylene hydrochlorination. This information guided the further optimization of the nitrogen-doped carbon catalyst.

### The active sites for acetylene in the SBMC catalysts

**Fig. 8:**
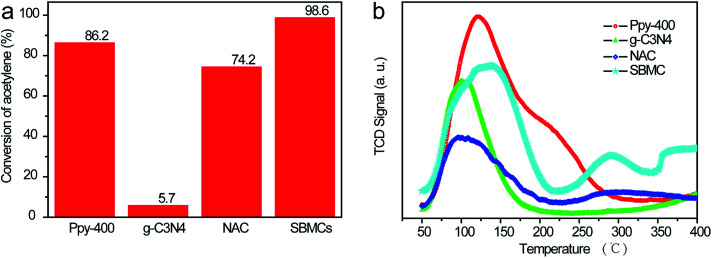
(a) Temperature programmed desorption data for acetylene and (b) catalytic performances of the catalysts. Reaction conditions: 200 °C, atmospheric pressure, space velocity of 1.0 mL g^−1^ min^−1^, HCl/C_2_H_2_ = 1.2/1.0 (volume ratio).

The active sites for acetylene in nitrogen-doped carbon catalysts during the hydrochlorination reaction remain unclear. Similarly, there are evidently three types of N species coexisting in the present SBMC catalysts. To investigate the effects of each single type of N species on the activation of acetylene, two model catalysts were synthesized: g-C_3_N_4_ and Ppy-400. The first represents an interesting synthetic carbon material with a total theoretical N content as high as 60.9% and an ideal structure containing only pyridinic and quaternary N, with no pyrrolic N species.^2^ The g-C_3_N_4_ was synthesized with urea as the precursor at 500 °C in a muffle furnace over a 3 h time span. Ppy contains only pyrrolic N, with no pyridinic and quaternary N. The formation of Ppy-400 with ppy as the precursor was performed at 400 °C in N_2_ for 2 h. As shown in Fig. 8a, although it had not been activated by forming pores, the Ppy-400 exhibited superior catalytic performance during acetylene hydrochlorination, with an acetylene conversion of 86.2%. This result clearly shows that pyrrolic N species served as the active sites and made a significant contribution to the activation of acetylene. In comparison, the g-C_3_N_4_ showed very poor activity for acetylene hydrochlorination, with a conversion of 5.7%, implying that the pyridinic and quaternary N species were not active. Fig. 8a demonstrates that, although they had almost the same specific surface area of ∼1000 m^2^ g^−1^,^1^ the SBMC exhibited excellent catalytic performance with 98.6% conversion. This value is much higher than that for the NAC (74.2%) mainly because of the higher N content, especially pyrrolic N species, in the SBMC. This study thus demonstrates that the presence and quantity of pyrrolic N species determines the catalytic activity of the SBMC for acetylene.

TPD is an effective technique providing a direct comparison of the adsorption and activation of reactants on different catalysts. This method was thus used to further elucidate the active sites for acetylene on the SBMC. The desorption temperature in the TPD profiles reflects the binding strength of the adsorbed species with the catalyst surface, while the peak area correlates with the amount of adsorbed species. As shown in Fig. 8b, the desorption temperature of ∼121 °C and the peak area in the TPD-C_2_H_2_ profile of the Ppy-400 were much higher than that for the g-C_3_N_4_. These results clearly show that the pyrrolic N species were able to adsorb and activate acetylene (Fig. 8a). In comparison, the desorption temperature of ∼101 °C and the peak area in the TPD-C_2_H_2_ profile of g-C_3_N_4_ were lower, indicating that carbon materials with pyridinic and quaternary N have less ability to adsorb acetylene than Ppy-400. However, although it had greater ability to adsorb acetylene than the NAC, the g-C_3_N_4_ showed very poor catalytic performance (Fig. 8a). This result demonstrates that carbon materials with pyridinic and quaternary N species will adsorb but not activate acetylene. In fact, pyridinic N and acetylene both donate electron pairs, and thus pyridinic N species would not be expected to adsorb acetylene. The g-C_3_N_4_ exhibited some ability to adsorb acetylene (Fig. 8b), attributed to the quaternary N species it contained. Thus, the three types of N species contributed to the activation of acetylene in the order: pyrrolic N > quaternary N > pyridinic N. The pyrrolic N species were therefore the critical active sites for the adsorption and activation of acetylene. As shown in Fig. 8b, a comparison of the TPD profiles of the SBMC and NAC reveals that the former exhibit higher adsorption and activation of acetylene, attributed to the higher concentration of pyrrolic N species in the SBMC. These data are in keeping with the results in Fig. 8a. A comparison of the TPD profiles for the SBMC and Ppy-400 indicates that the Ppy-400 exhibited greater adsorption and activation of acetylene as a result of a higher level of pyrrolic N species. Even so, although it showed many more active sites for acetylene, the Ppy-400 had lower catalytic performance than the SBMC. This result is attributed to the lower amount of pyridinic N species in the Ppy-400. The pyridinic N species adsorbed and activated hydrogen chloride, which was the rate-limiting step for acetylene hydrochlorination over the SBMC catalyst. Consequently, this result further demonstrates that there were two active sites in the SBMC, pyridinic N and pyrrolic N, acting as the active sites for hydrogen chloride and acetylene, respectively.

**Fig. 9:**
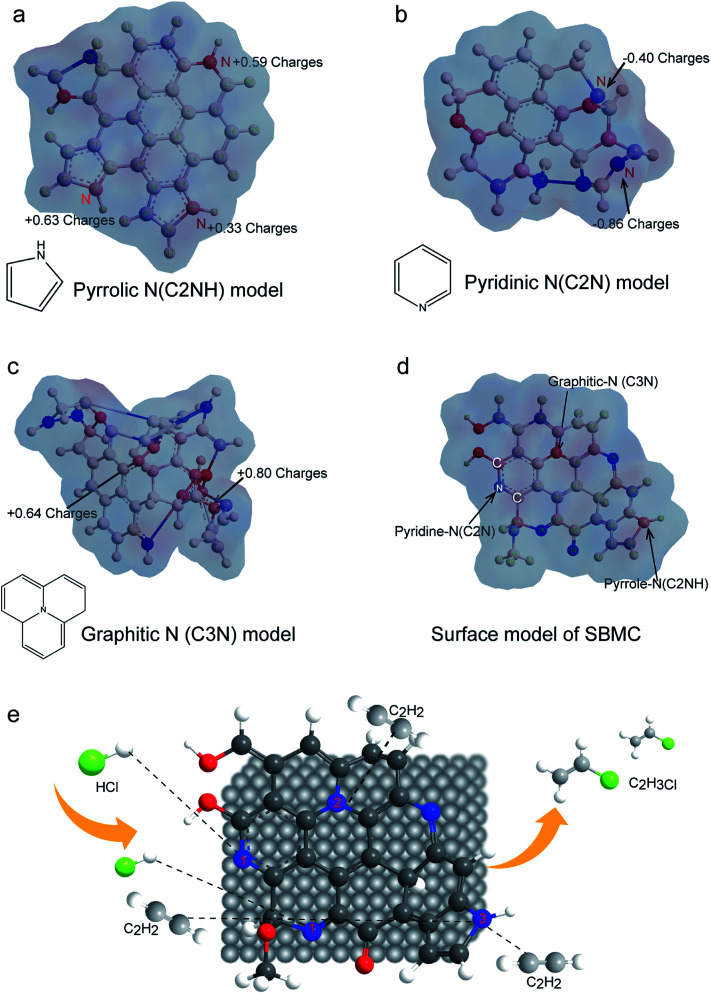
A diagram showing the electron cloud distributions on the surfaces of model catalysts with only (a) pyrrolic N species, (b) pyridinic N species, (c) graphitic N species, (d) and with all three types of N species. (e) A diagram of the hydrochlorination of acetylene on the SBMC catalyst.

In a typical catalytic reaction, the electron distribution at the active sites polarizes the reactant molecules on the surface and reduces the reaction activation energy in order to increase the reaction rate. To further investigate the nature of the three types of N species on the SBMC with regard to activating acetylene, the electron distributions of the catalysts are simulated in Fig. 9. In Fig. 9a, the pyrrolic N species show large positive charges. During the acetylene hydrochlorination, acetylene is the electron donor, and thus it tends to adsorb on the pyrrolic N model catalyst in keeping with the TPD-C_2_H_2_ data and the catalytic performance of the ppy-400 (Fig. 8). Fig. 8b demonstrates that the pyridinic N species are negatively charged. Hydrogen chloride will be readily polarized by the negative charge cloud, and thus it will be adsorbed onto pyridinic N species. This explains why pyridinic N species are the active sites for hydrogen chloride. As shown in Fig. 9c, there is a positive charge on each graphitic N, and therefore these tend to adsorb acetylene, in agreement with the TPD-C_2_H_2_ results for g-C_3_N_4_ (Fig. 8b). However, in a catalytic reaction, the adsorption of reactant molecules is not the same as activation. Thus, the g-C_3_N_4_ catalyst demonstrated very poor catalytic performance during acetylene hydrochlorination (Fig. 8a). The theoretical electron cloud distributions on the model catalysts were highly consistent with the TPD-C_2_H_2_ data and catalytic performances of these materials (Fig. 8). The study of the electron distributions confirmed that the different N species can activate acetylene, which explains the performance of the N-doped carbon catalyst and is in agreement with previous reports from Hutchings' group. Hutchings determined that the catalytic activity of a metal chloride for acetylene gradually increased with the metal ion potential.^10,57^ Schematic diagrams of the three types of N species distribution on the SBMC catalyst are presented in Fig. 9d while a diagram of the acetylene hydrochlorination mechanism on the SBMC catalyst is shown in Fig. 9e. Initially, the hydrogen chloride molecule is polarized and adsorbed onto the pyridinic N species. In addition, acetylene is adsorbed onto the positively charged sites such as pyrrolic N and then polarized. Secondly, the polarized hydrogen chloride reacts with the polarized acetylene to synthesize the VCM. Finally, the VCM leaves to complete the hydrochlorination.

## Conclusions

This work produced a unique, green, low-cost and metal-free catalyst *via* the carbonization of SBM with ZnCl_2_ at 500–700 °C. These materials exhibited superior catalytic performance during acetylene hydrochlorination over a time span of 110 h, with an initial acetylene conversion of over 99% and 98% selectivity for VCM at 200 °C. XPS, TPD and catalytic activity evaluations established that pyridinic and pyrrolic species represent the active sites for hydrogen chloride and for acetylene, respectively. Charge calculations based on model catalysts indicated that the activity of the pyrrolic species contributed to the potential of the SBMC catalysts. Investigation of the mechanism for acetylene hydrochlorination over these materials established that the N-doped catalyst had two different active sites that worked to adsorb and activate hydrogen chloride and acetylene, in contrast to metal chloride catalysts such as HgCl_2_. These SBMC have the potential to serve as substitutes for HgCl_2_/AC as catalysts for VCM production. The results of this work also suggest the feasibility of using biomass as a feedstock to synthesize functional carbon materials for acetylene hydrochlorination. The mechanistic details reported herein also suggest new approaches to further optimization, specifically, by increasing the concentration of pyridinic moieties in order to promote the adsorption and activation of hydrogen chloride. Finally, these results are expected to encourage further experimentation to improve N-doped catalysts in order to enhance the adsorption and activation of acetylene by increasing the level of pyrrolic species and/or doping with additional heteroatoms.

## Experimental

### Materials

AC (neutral, coal-based carbon) was purchased from the Ningxia Guanghua Activated Carbon Co., Ltd. SBM was obtained from the Yihai Kerry Co., Ltd. The reagents, including ZnCl_2_ (AR), acrylamide (AR) and concentrated hydrochloric acid (>36%, AR) were purchased from the Taitan Technology Co., Ltd. The gases, including acetylene (99.99%) and nitrogen (99.99%), were purchased from the Shanghai Lvming Gas Co., Ltd. Hydrogen chloride (99.998%) was purchased from the Beijing Lvling Gas Co., Ltd.

### Catalyst preparation

SBM was employed as the source of carbon, nitrogen and oxygen, with ZnCl_2_ as the activation agent. SBMC was prepared by first drying the SBM at 80 °C for 24 h, followed by grinding to a 20–40 mesh particle size. The SBM was subsequently impregnated by immersion in a ZnCl_2_ solution, using the incipient wetness impregnation technique, with an ZnCl_2_/SBM mass ratio of approximately 1 : 1. The SBM was subsequently dried at 120 °C for 6–10 h and the dried material was calcined at 500, 600, 700 or 800 °C to generate nitrogen-doped porous carbon or SBMC. The product was obtained by washing the crude material to remove excess ZnCl_2_, using 2 N HCl, deionized water and ethanol. The resulting materials were termed SBMC-500, SBMC-600, SBMC-700 and SBMC-800 according to the carbonization temperature. Similarly, the term SBMC-*x*ZnCl_2_-600 refers to a material in which *x* is the ZnCl_2_/SBM ratio. NAC catalysts were prepared by an incipient wetness impregnation technique using AC as the carrier and acrylamide as the N precursor. The mixture of AC and acrylamide was dried at 120 °C for 6–12 h after which the dried mixture was calcined at 650 °C for 4 h under N_2_ to obtain the final product. M_*x*_Cl_*y*_@NAC catalysts were prepared using metal chlorides (M_*x*_Cl_*y*_) as the active components and NAC as the carrier, employing a simple incipient wetness impregnation technique.

### Catalyst characterization

Brunauer–Emmett–Teller (BET) surface area data were collected by obtaining nitrogen adsorption isotherms at 77 K using a Micromeritics ASAP 2020 analyser (ASAP 2020, Micromeritics Company, USA). Wide-angle XRD patterns (10°–90° over 2 h) were collected using a Bruker D8 Advanced X-ray diffractometer with Cu Kα radiation (*k* = 1.5406 Å) at 40 kV and 40 mA (Bruker D8, Brucker Company, Germany). TEM images were acquired with a JEM 2010 electron microscope at an accelerating voltage of 200 kV to examine sample morphologies (JEM-2010, JEOL Company, Japan), and field emission scanning electron microscope (S-4800, Hitachi Company, Japan). XPS data were generated using an Axis Ultra spectrometer with monochromatized Al Kα X-ray radiation as the excitation source (225 W) (EscaLab 250Xi, Thermo Fisher Scientific Company, USA). Raman microspectroscopy was performed with a Renishaw InVia unit having an Ar ion laser (inVia reflex, Renishaw Company, UK). TGA data were acquired using a TA instrument, operating under air at a flow rate of 100 mL min^−1^. The temperature of each sample was increased from 30 to 800 °C at a heating rate of 10 °C min^−1^ (SDT Q600, TA Company, USA). TPD trials were carried out with a Micromeritics ASAP 2720 instrument with a temperature ramp of 40–650 °C, a ramp rate of 10 °C min^−1^ and a flow of 45 mL min^−1^ (ASAP 2720, Micromeritics Company, USA).

### Catalytic reactions

Catalytic performance was evaluated in a fixed-bed reactor (i.d. 6 mm), the temperature of which was regulated using a temperature controller (Y-Feng, Yefeng Company, China). In each trial, a 1.0 g sample of catalyst was placed in the reactor and nitrogen was used to purge water and air before starting the reaction. Hydrogen chloride gas was passed through the reactor at a flow rate of 20 mL min^−1^ to activate the catalyst. Thereafter, acetylene (1.0 mL min^−1^) and hydrogen chloride (1.15 mL min^−1^) were fed through the heated reactor at a gas hourly space velocity (GHSV) of 30 h^−1^ and a reaction temperature of 200 °C. The reaction products were analysed by gas chromatography (GC-2014, Shimadzu Company, Japan).

The conversion of acetylene was calculated as:
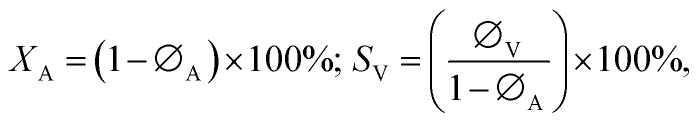
where *X*_A_ is the conversion of acetylene, *S*_V_ is the selectivity for VCM, ∅_A_ is the ratio of the peak area of acetylene and ∅_V_ is the ratio of the peak area of VCM.

The space-time yield (STY) of VCM was calculated as in the following equation,
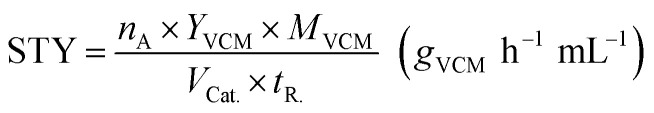
where *n*_A_ represents the moles of acetylene in the raw gas (mol), *Y*_VCM_ represents the yield of VCM, *M*_VCM_ represents the molar mass of VCM (62.5 g mol^−1^), *V*_Cat._ represents the volume of catalyst in reactor (mL), *t*_R._ represents reaction time (h).

## Conflicts of interest

There are no conflicts to declare.

## Supplementary Material

RA-010-D0RA00475H-s001
