# Supplementary material for: Nitrogen-doped porous carbon from biomass with superior catalytic performance for acetylene hydrochlorination
Source: RSC Adv. 2020 Apr 9;10(25):14556–69. doi: 10.1039/d0ra00475h (PMC9051911; doi:10.1039/d0ra00475h)
Supplement: RA-010-D0RA00475H-s001 [file RA-010-D0RA00475H-s001.pdf]

Electronic Supplementary Material (ESI) for RSC Advances.
This journal is © The Royal Society of Chemistry 2020

A Nitrogen-Doped Porous Carbon from Biomass with Superior Catalytic Performance for Acetylene Hydrochlorination

Zhaobing Shen,[a, b] Yue Liu,[b] Yejun Han,[b] Yejun Qin,[a] Jinhua Li,[a] Ping Xing,[a] Biao Jiang* [a, b]

a. Shanghai Green Chemical Engineering Research Centre, Shanghai Institute of Organic Chemistry, Chinese Academy of Sciences, No. 345 Lingling Road, Shanghai, P. R. China.
b. Green Chemical Engineering Research Centre, Shanghai Advanced Research Institute, Chinese Academy of Sciences, No. 99 Haike Road, Zhangjiang Hi-Tech Park, Pudong Shanghai, P. R. China

E-mail: jiangb@sioc.ac.cn

Reference	Catalyst	T (℃)	GHSV[a] (h^{-1})	$X_{acetylene}$[b] (%)	S_{VCM}[c] (%)	S_{BET}[d] (m^2 g^{-1})	N[e] (wt %)	STY[f] (g_{VCM} h^{-1} mL^{-1})	STY/(S_{BET} g_N)[g] (g_{VCM} h^{-1} mL^{-1} m^{-2} g^{-1})
1	B,N-G	150	36	80	98	N	10.9	0.08	N
2	g-C3N4@AC	180	50	65	98	874	8.6	0.09	1.19E-03
3	PANI-doped carbon	180	36	72	98	592	1.9	0.07	6.32E-03
4	MOF-doped carbon	180	50	80	98	1665	1.6	0.11	4.12E-03
5	N-OMC	180	50	34	98	613	3.9	0.05	1.95E-03
6	AC-n-U500	180	30	75	98	1082	4	0.06	1.43E-03
7	S, N doped carbon	180	50	80	98	49.1	7.9	0.11	2.83E-02
8	TPPB@SAC	180	50	83	98	N	N	0.11	N
9	Si@N-C	200	30	80	98	281	7.1	0.07	3.30E-03
10	p-BN	200	40	72	96	N	N	0.08	N
11	TPPB@SAC	200	30	92	98	873	N	0.08	N
12	PAN-derived catalyst	200	30	75	98	12	18.5	0.06	2.78E-02
13	g-C3N4	200	30	90	98	47.9	29.4	0.07	5.26E-03
14	N-MC-G	220	30	80	98	582	4.2	0.07	2.69E-03
15	ZIF-8/SAC	220	30	80	98	1058	1.1	0.07	5.66E-03
16	NPC	220	1.7[h]	85	98	443	2.5	N	N
17	Defective N-doped carbon	220	36	80	98	295	2.4	0.08	1.12E-02
18	ND@G	220	30	85	98	382	N	0.07	N
19	NR@CAC	220	30	96	98	703	3.4	0.08	3.31E-03
20	D-AC-M	220	30	60	98	1191	1.9	0.05	2.18E-03
21	NC-2	220	50	97	98	301	9.7	0.13	4.56E-03
22	PSAC-N	250	30	90	98	936	4.9	0.07	1.62E-03
23	13X zeolite	320	60	85	98	501		0.14	N
**This work**	SBMC	180	50	80	98	1038	6.9	0.11	1.53E-03
	SBMC	200	50	87	98	1038	6.9	0.12	1.67E-03
	SBMC	220	50	95	98	1038	6.9	0.13	1.82E-03

[a] C_2H_2 GHSV; [b] conversion of acetylene; [c] selectivity for VCM; [d] the specific area of catalysts; [e] nitrogen atom content on the catalysts by XPS; [f] the space-time yield of VCM; [g] the value of STY was normalized by the surface area and nitrogen contents; [g] the GHSV is 1.7 mL g^{-1} h^{-1}.

Table S1. Comparation of catalytic performance among the previous works and SBMC

Table S2

Reference	Catalyst	T (℃)	X_A[a] (%)	X_A[b] (%)	t[c] (h)	deactivation rate[d] (%)
1	B,N-G	150	95	80	2	7.500
2	g-C3N4@AC	180	75	65	7	1.429
3	PANI-doped carbon	180	76	72	9	0.444
4	MOF-doped carbon	180	60	46	50	0.280
5	N-OMC	180	35	30	20	0.250
6	AC-n-U500	180	22	21	20	0.050
7	S, N doped carbon	180	82	78	9	0.444
8	TPPB@SAC	180	84	81	40	0.075
9	Si@N-C	200	82	80	100	0.020
10	p-BN	200	100	95	1000	0.005
11	TPPB@SAC	220	97	94	300	0.010
12	PAN-derived catalyst	280	88	82	200	0.030
13	g-C3N4	220	94	92	40	0.050
14	N-MC-G	220	80	80	100	0.000
15	ZIF-8/SAC	220	81	76	150	0.033
16	NPC	260	96	85	20	0.550
17	Defective N-doped carbon	220	96	75	14	1.500
18	ND@G	220	82	80	200	0.010
19	NR@CAC	220	95	92	200	0.015
20	D-AC-M	220	60	60	90	0.000
21	NC-2	220	98	88	50	0.200
22	PSAC-N	250	70	58	160	0.075
23	13X zeolite	320	95	77	6	3.000
**This work**	SBMC	200	99	96	110	**0.027**

[a] the initial conversion of acetylene; [b] the final conversion of acetylene; [c] reaction time; [d] the deactivation rate of the catalysts.

Comparation of catalytic stability among the previous works and SBMC
